# Improvement in the Safety of Rapid Sequence Intubation in the Emergency Department with the Use of an Airway Continuous Quality Improvement Program

**DOI:** 10.5811/westjem.2019.4.42343

**Published:** 2019-06-03

**Authors:** John C. Sakles, Cassidy C. Augustinovich, Asad E. Patanwala, Garrett S. Pacheco, Jarrod M. Mosier

**Affiliations:** * University of Arizona College of Medicine, Department of Emergency Medicine, Tucson, Arizona; † Virginia Commonwealth University School of Medicine, Richmond, Virginia; ‡ University of Sydney, Faculty of Medicine and Health, Sydney, Australia; ¶ University of Arizona College of Medicine, Department of Medicine, Division of Pulmonary, Allergy, Critical Care, and Sleep Medicine, Tucson, Arizona

## Abstract

**Introduction:**

Airway management in the critically ill is associated with a high prevalence of failed first attempts and adverse events which negatively impacts patient care. The purpose of this investigation is to describe an airway continuous quality improvement (CQI) program and its effect on the safety of rapid sequence intubation (RSI) in the emergency department (ED) over a 10-year period.

**Methods:**

An airway CQI program with an ongoing airway registry was initiated in our ED on July 1, 2007 (Academic Year 1) and continued through June 30, 2017 (Academic Year 10). Data were prospectively collected on all patients intubated in the ED during this period using a structured airway data collection form. Key data points included method of intubation, drugs and devices used for intubation, operator specialty and level of training, number of intubation attempts, and adverse events. Adult patients who underwent RSI in the ED with an initial intubation attempt by emergency medicine (EM) resident were included in the analysis. The primary outcome was first pass success which was defined as successful tracheal intubation with a single laryngoscope insertion. The secondary outcome was the prevalence of adverse events associated with intubation. Educational and clinical interventions were introduced throughout the study period with the goal of optimizing these outcomes. Data were analyzed by academic year and are reported descriptively with 95% confidence intervals (CI) of the difference of means.

**Results:**

EM residents performed RSI on 342 adult patients during Academic Year 1 and on 445 adult patients during Academic Year 10. Over the 10-year study period, first pass success increased from 73.1% to 92.4% (difference = 19.3%, 95% CI 14.0% to 24.6%). The percentage of patients who experienced an adverse event associated with intubation decreased from 22.5% to 14.4% (difference = −7.9%, 95% CI −13.4% to −2.4%). The percentage of patients with first pass success without an adverse event increased from 64.0% to 80.9% (difference = 16.9%, 95% CI 10.6% to 23.1%).

**Conclusion:**

The use of an airway CQI program with an ongoing airway registry resulted in a substantial improvement in the overall safety of RSI in the ED as evidenced by an increase in first pass success and a decrease in adverse events.

## INTRODUCTION

Critically ill patients frequently require airway management in the emergency department (ED) and this is usually accomplished with rapid sequence intubation (RSI).^1^ Emergency airway management has been shown to be associated with a high prevalence of failed first attempts and adverse events, both of which can negatively affect patient care.^2^,^3^,^4^,^5^,^6^,^7^,^8^,^9^,^10^,^11^ Multiple intubation attempts are associated with an increase in adverse events, many of which can be life-threatening.^3^,^12^,^13^ When adverse events occur during emergency intubation, the effect on patient outcomes are much more severe compared to elective intubation.^9^,^14^ To maximize the safety of intubation in the ED, the goal should be to achieve first pass success without any adverse events. Without measuring important outcomes like first pass success and adverse events, it is difficult to improve patient care with emergency airway management. In our ED we developed an airway continuous quality improvement (CQI) program that incorporated an ongoing airway registry with the aim of improving the safety of emergency airway management. This study describes our airway CQI program and its associated effect on the safety of airway management in our ED.

## METHODS

### Study Design and Setting

This study was conducted at an urban academic ED with a Level 1 trauma center and includes data over the 10-year period from July 1, 2007 (Academic Year 1) to June 30, 2017 (Academic Year 10). Since this project was a CQI initiative it was granted an exemption by the University of Arizona Institutional Review Board.

This institution serves as the training site for two Accreditation Council for Graduate Medical Education (ACGME) accredited three-year emergency medicine (EM) residency programs, one of which is a university hospital-based program and the other which is a community-hospital based program. In addition, the institution supports a five-year combined emergency medicine/pediatrics (EM/PEDS) residency program. There are currently a total of 79 residents in these three training programs. Intubations in this ED are performed primarily by the EM and EM/PEDS residents under direct supervision by the EM attending.

Throughout the study period, multiple airway devices were stocked in the ED, with clinical availability of the devices varying somewhat. The availability of these devices were based on hospital considerations and manufacturer issues and were not the result of the CQI program. The direct laryngoscope (DL) with Macintosh and Miller blades was available in the ED throughout entire study period. One or more video laryngoscopes (VL) were also available throughout the study period. The hyperangulated GlideScope® was available from Academic Year 1 continuously through the second month of Academic Year 10, and the standard geometry GlideScope® (MAC T3 and T4) was available from Academic Year 8 continuously through the second month of Academic Year 10. The standard geometry C-MAC® was available from the eighth month in Academic Year 3 continuously through Academic Year 10 and the hyperangulated C-MAC® (D-blade) was available from the tenth month in Academic Year 4 continuously through Academic Year 10. Other VLs such as the McGrath®, the Pentax Airway Scope® and the Res-Q-Scope® were available sporadically on a trial basis for brief periods of time. Rescue devices available during the study period included a supraglottic device (LMA Fastrach®) and a commercially available surgical airway kit (Cook Universal Emergency Cricothyrotomy Catheter Set®).

**Population Health Research Capsule**

What do we already know about this issue?

Airway management is frequently performed in the emergency department (ED) and is associated with a high prevalence of adverse events.

What was the research question?

Could monitoring of airway performance in conjunction with an airway continuous quality improvement (CQI) program improve the safety of rapid sequence intubation in the ED?

What was the major finding of the study?

Over a 10-year period the airway CQI program was associated with a 19.3% absolute increase in first pass success and a 7.9% absolute decrease in adverse events.

How does this improve population health?

By monitoring airway management practices in the ED and making iterative clinical adjustments, the safety of this high-risk procedure can be improved.

This study included all adult patients 18 years of age and older that underwent RSI in the ED by an EM resident as the first operator.

### Airway CQI Program and Airway Registry

The airway CQI program and airway registry was started on July 1, 2007. A single page (double sided) paper-based airway data collection form was developed to capture important clinical information regarding intubations in the ED (Appendix: https://escholarship.org/uc/item/8tv7v5nz#supplemental). The form was housed in the physician charting area of the ED. After each intubation the resident was expected to fill out the airway form. The senior investigator reviewed all airway forms on a weekly basis and cross-referenced them with the electronic medical record and the hospital admission log. If any intubations were identified without a corresponding airway form, a blank form was given to the resident for completion. If any of the airway forms had incomplete or contradictory information, the resident was interviewed by the senior author and the form updated as appropriate. Timeliness of completion of the airway forms was recorded during the last two years of the study period. 70% of the forms were filled out on the day of the intubation, 86% were filled out within two days of the intubation, and 94% were filled out within seven days of the intubation. Ultimately, 100% of airway forms were completed.

The airway form was designed to capture important information about the patient, the operator and the characteristics of the procedure. This included data such as patient age and sex, diagnosis, operator post-graduate year (PGY), operator specialty, reason for intubation, method of intubation, difficult airway characteristics, drugs used for intubation, device used on each attempt, outcome of each attempt, and adverse events associated with intubation. Adverse events and their definitions are presented in Table 1. An intubation attempt was defined as the insertion of the laryngoscope blade into the mouth of the patient, regardless of whether an attempt was made to insert a tracheal tube. First pass success was defined as successful tracheal intubation on a single laryngoscope insertion. First pass success without an adverse event was defined as successful tracheal intubation on the first attempt without the occurrence of any adverse events.

The airway training program for the EM and EM/PEDS residents consisted of multiple educational components. Residents in the university-based EM and EM/PEDS program complete a four-week rotation on the anesthesia service to learn fundamental aspects of airway management. Residents in the community-based EM program do not participate in an anesthesia rotation. During the intern year there is a full day airway orientation that includes three hours of didactics and three hours of hands-on experience in the simulation laboratory. Each year there is a difficult airway lab in the simulation lab. The difficult airway lab has been expanded over the study period and currently includes the following six hands-on stations: 1. Rigid laryngoscopy including both DL and VL, 2. Flexible laryngoscopy, 3. Face mask ventilation and supraglottic insertion, 4. Surgical airway techniques, 5. Pediatrics, and 6. Ultrasound assessment of the airway and hemodynamics. Airway lectures and cases are presented on an ongoing basis at the regularly scheduled conference time throughout the academic year. Based upon ongoing data analysis from the airway registry, these educational programs have been modified and expanded to address varying clinical concerns. For example, use of a VL for the initial attempt has been increasingly promoted over the entire study period due to the beneficial effects observed with the CQI program. Even if DL was the primary approach to be performed, use of a standard geometry VL was strongly encouraged.^15^ In the last four years of the study, heavy emphasis was placed on the importance of physiologic optimization prior to intubation.^16^ Appropriate preoxygenation strategies were emphasized and non-invasive ventilation (NIV) for hypoxemic patients with shunt physiology was encouraged.^17^ The use of apneic oxygenation and high flow nasal oxygen (HFNO) was also encouraged for patients at high risk of oxygen desaturation. Hemodynamic optimization before intubation, with crystalloids, blood products, vasopressors or inotropes, as clinically indicated, was also strongly emphasized. Pre-intubation point-of-care cardiovascular ultrasonography was promoted as a means to identify patients at risk of hemodynamic collapse and to allow proactive management with appropriate resuscitation strategies. Operators were encouraged to use their assessment of cardiac function and evaluation of the inferior vena cava (IVC) to determine the need for intravenous fluids or vasoactive drugs in the peri-intubation period.

### Outcome Measures

The primary outcome measure was first pass success. The secondary outcome measure was the number of patients who experienced an intubation associated adverse event. Other important variables such as first pass success without an adverse event and procedural characteristics are reported as well.

### Data Analysis

Data from the collected paper forms was entered into Excel® for Windows 2013 (Microsoft, Redmond, Washington) and transferred in to STATA 13® (StataCorp, College Station, Texas) for analysis. Data was analyzed by academic year (July 1, 2007–June 30, 2017). The first year of the study was the academic year 2007–2008 (Academic Year 1), and the last year of the study was academic year 2016–2017 (Academic Year 10). Data are reported descriptively with 95% confidence intervals (CIs) or the 95% CI of the difference of the means, as appropriate.

## RESULTS

### Characteristics of Study Subjects

There were 5,229 total intubations performed in the ED over the 10-year study period. Of these, 4,362 were performed using an RSI technique. The number of adult patients who underwent RSI by an EM resident was 3,763, and these were included in this analysis. The mean age in years of patients intubated was 46.1 (range 18–98). 1,307 (34.7%) were women and 1,431 (38.0%) were trauma patients.

### First Pass Success

First pass success by academic year is listed in Table 2. Over the 10-year period, first pass success increased from 73.1% to 92.4% (difference = 19.3%, 95% CI 14.0% to 24.6%). Intubation was successful within two attempts in 88.6% of patients in Academic Year 1 and 99.3% of patients in Academic Year 10. Intubation was successful within three attempts in 94.7% of patients in Academic Year 1 and in 100% of patients in Academic Year 10.

### Adverse Events

The number of patients who experienced an adverse event are listed in Table 3. Over the 10-year period, the percentage of patients who experienced an adverse event decreased from 22.5% to 14.6% (difference = −7.9%, 95% CI −13.4% to −2.4%).

Specific adverse events are listed in Table 4. Hypoxemia (SpO_2_<90%) was the most common adverse event and occurred in 76.6% (59/77) of the patients with adverse events in Academic Year 1 and in 76.9% (50/65) of the patients with adverse events in Academic Year 10. The percentage of patients who had hypoxemia decreased from 17.3% to 11.2% (difference = −6.0%, 95% CI −11.0% to −1.1%).

Complete oxygen saturation data was available in 73.4% (251/342) of patients in Academic Year 1 and in 86.3% (384/445) of patients in Academic Year 10. Oxygen desaturation to <80% occurred in 8.8% of patients in Academic Year 1 and in 3.6% of patients in Academic Year 10 (difference = −5.2%, 96% CI −8.7% to −1.7%). Oxygen desaturation to <70% occurred in 5.0% of patients in Academic Year 1 and in 1.6% of patients in Academic Year 10 (difference = −3.4%, 95% CI −6.0% to −0.8%).

The percentage of patients who had a recognized esophageal intubation decreased from 4.4% in Academic Year 1 to 0% in Academic Year 10 (difference = −4.4%, 95% CI −6.4% to −2.2%).

### First Pass Success without an Adverse Event

First pass success without an adverse event increased over the 10-year period from 64.0% to 80.9% (difference = 16.9%, 95% CI 10.6% to 23.1%).

### Procedural Characteristics

Procedural characteristics are listed in Table 5. VL use on the initial attempt increased from 44.7% to 97.8% (difference = 53.0%, 95% CI 47.6% to 58.5%) over the 10-year period. GlideScope ® use decreased from 86.9% in Academic Year 1 to 5.5% in Academic Year 10 and C-MAC® use increased in Academic Year 1 from 0% to 94.5% in Academic Year 10, reflecting clinical availability of the two devices. In Academic Year 9, when the GlideScope® and C-MAC® were both available, clinical use was very similar (GlideScope® 48.6% and C-MAC® 51.4%).

Use of succinylcholine increased from 40.4% to 53.9% (difference = 13.6%, 95% CI 6.6% to 20.5%) and use of ketamine increased from 2.0% to 14.6% (difference = 12.6%, 95% CI 9.0% to 16.2%). Senior residents (PGY ≥3) performed 43.6% of the intubations in Academic Year 1 and 51.2% in Academic Year 10 (difference = 7.7%, 95% CI 6.6% to 14.7%). The need for a device switch after a failed intubation attempt decreased from 17.8% to 0.5% (difference = −17.4%, 95% CI −21.5% to −13.3%), and the need for an EM attending to rescue an EM resident decreased from 5.8% to 0.2% (difference = −5.6%, 95% CI −8.1% to −3.1%).

Over the entire study period there were 6 adult patients (0.16%) with a failed RSI by an EM resident that ultimately went on to receive a surgical airway (one in Academic Year 2, one in Academic Year 5, two in Academic Year 6, one in Academic Year 7, and one in Academic Year 9).

## DISCUSSION

Airway management in the critically ill is known to be associated with a high prevalence of failed intubation attempts and serious adverse events, which can negatively impact patient care. To maximize patient safety, the goal of airway management in the ED should be first pass success without adverse events. Just over a decade ago we developed an airway CQI program with an ongoing airway registry to monitor our airway performance in the ED. We found over the 10-year period that first pass success increased from 73% to 92% and adverse events decreased from 23% to 15%. Patients that had first pass success without an adverse event increased by 17%. While it is difficult to say exactly which components of our program were responsible for this improvement, we believe there were a couple of key factors. One is the adoption of near universal VL use for initial intubation attempts. Over the 10-year period VL use increased from 45% to 98%. Since the operators in this study were EM residents, they had variable and limited experience with intubation. When using a DL, the supervising attending had virtually no ability to assist the resident with laryngoscopy and identification of the airway, thus increasing the risk of a failed intubation attempt or esophageal intubation. On the other hand, when using a VL, the EM attending could see everything the resident was seeing during laryngoscopy and thus could assist with navigation to and identification of the airway. This would have the effect of improving first pass success and avoiding inadvertent misplaced tubes in the esophagus. This notion is supported by the fact that with essentially universal VL use, the esophageal intubation rate fell to 0% and the need for the EM attending to rescue the resident fell to 0.2%. The CQI program allowed us to realize the beneficial effect of VL in our ED and thus was largely responsible for the promotion of increased VL use.

The other factor that we believe played an important role in the improvement we observed was greater emphasis on pre-intubation physiologic optimization during the latter half of the study period.^16^ Greater attention was given to optimization of preoxygenation and hemodynamics before intubation. Appropriate preoxygenation methods were emphasized to maximize oxygen stores before RSI.^17^ NIV was encouraged for preoxygenation in patients with shunt physiology who remained hypoxemic with conventional preoxygenation techniques.^18^ The use of apneic oxygenation and HFNO was encouraged for patients at great risk of oxygen desaturation. Point-of-care ultrasound was encouraged prior to intubation to evaluate cardiovascular function and to guide decision making for appropriate hemodynamic support in the peri-intubation period.

There were some minor changes in procedural characteristics over the 10-year period. There was a small increase (8%) in the number of intubations performed by senior residents. This may also have contributed to the increase in first pass success observed in the study, as previous work in our ED has demonstrated an increase in first pass success with VL with increasing post-graduate level.^19^ Other notable differences over the study period involved the use of pharmacologic agents for RSI. Succinylcholine use increased by 14%, likely representing a shift away from the use of long acting neuromuscular blocking agents in trauma patients with traumatic brain injuries to allow ongoing neurologic assessment. Ketamine use increased by 13%, likely reflecting an increased awareness of ketamine’s safety in head injured patients and its beneficial hemodynamic profile in shocked patients. Based on previous studies it is unlikely that any of these pharmacologic changes we observed would have significantly affected first pass success.^20^,^21^

Many other programs have also attempted to improve the safety of emergency airway management with a variety of clinical interventions. In a university affiliated ED, Hwang and colleagues reported on their experience with an airway CQI initiative over a three-year period.^22^ With procedural standardization, airway education and equipment preparation they found that they were able to increase their first pass success from 68% to 79%. They also observed a decrease in adverse events from 16% to 8%. It is of interest that in their study, they also had a significant increase in VL use for first intubation attempts, from 9% in year 1 to 60% in year 3. In an academic pediatric ED, Kerrey and colleagues instituted a CQI program with the goal of reducing the prevalence of oxygen desaturation during emergency intubation.^23^ With the use of an RSI checklist, the use of VL, and the restriction of intubation to specific providers, they observed a reduction of intubation associated hypoxemia from a historical control of 33% to 16%. In the intensive care unit (ICU) setting, Jaber and colleagues instituted an intubation bundle in an attempt to decrease complications associated with emergency intubation.^24^ Their intubation bundle had 10 components and included such things as pre-intubation fluid loading, early vasopressor use, and NIV for preoxygenation. They found a reduction in life-threatening complications from 34% to 21% in the six-month period after the introduction of this bundle. Mayo and colleagues reported on their results from a CQI program in their intensive care unit (ICU), where emergency intubations were performed by pulmonary/critical care fellows.^25^ Their CQI program incorporated a combined team approach, mandatory checklist use, crew resource management techniques and scenario-based training using computerized patient simulators. Over a three-year period, they observed a first pass success of 62%. Of note, their standard approach for emergency intubation was not RSI, but rather sedative only intubation. In the prehospital setting, Olvera and colleagues reported on their experience with the introduction of a CQI program in a large aeromedical transport service.^26^ They implemented an elaborate CQI program across 160 helicopter bases and over a three-year period found an improvement in first pass success from 85% to 95%. First pass success without desaturation was found to increase from 84% to 94%. In another prehospital study, Jarvis and colleagues described their experience with an intubation bundle designed to reduce the occurrence of peri-intubation hypoxemia.^27^ Their intubation bundle included goal directed preoxygenation, apneic oxygenation, and the use of delayed sequence intubation. They found that implementation of this bundle in their ground ambulance service resulted in a decrease in intubation associated hypoxemia from 44% to 3.5%. As a whole, the results of these studies consistently show a temporal relationship between monitoring airway performance with iterative clinical adjustments and improved procedural safety.

### Limitations

There are several important limitations in this study. First, this is single center observational study at an academic center where EM residents perform most of intubations in the ED, so these results might not be generalizable to EDs with fully trained practitioners at non-teaching hospitals. In particular, the value of VL may be overstated when extrapolated to these clinicians and environments. Second, the data collected was all self-reported by the operator and collected at varying times after the intubation. It is thus highly subject to recall bias. Studies on emergency airway management have demonstrated that operators tend to over-report first pass success and under-report adverse events in the chart compared to what is found on recorded videos of the intubation.^28^,^29^ In one study it was found that intubation associated desaturation events were only documented in the chart in 16% of the cases, when video review of those same cases identified a 33% prevalence of oxygen desaturation.^29^ While video review of ED intubations would be ideal to obtain the highest quality data, this was not possible given the structure of our ED. Additionally, video review is expensive and very labor intensive, and while important for research projects, it is simply impractical for widespread adoption of airway CQI programs in the ED. Third, the definitions used for adverse events in our study were not very specific and could be subject to varying interpretation by the operators. For example, if hypotension or a cardiac arrest occurred shortly after intubation, the operator may not believe or document this as a complication of the procedure. Finally, numerous educational and clinical changes were made throughout the 10-year study period, so it is impossible to ascertain which of these interventions were responsible for the clinical improvements we observed. While increased use of VL and greater emphasis on physiologic optimization appear to be associated with the improvement we have observed, this is not evidence of causation.

## CONCLUSION

To maximize the safety of airway management in the ED, the goal should be first pass success without adverse events. We developed an airway CQI program in our ED that incorporated an ongoing airway registry to monitor and improve the safety of emergency intubation. With this approach we were able to substantially increase first pass success and decrease adverse events associated with intubation. We recommend that all EDs monitor their airway performance and attempt to improve upon it so that patent safety can be maximized.

## Supplementary Information



## Figures and Tables

**Table 1:** Definitions of adverse events.

Adverse event	Definition
Aspiration	Presence of vomit at the glottic inlet visualized during intubation in a previously clear airway
Cardiac arrest	Pulseless dysrhythmia occurring during intubation
Cuff leak	Air leak around a cuffed ETT requiring replacement of the ETT
Dental trauma	Fracture or avulsion of tooth during intubation
Dysrhythmia	Bradycardia or any ventricular dysrhythmia during intubation
Esophageal intubation	Inadvertent placement of the ETT in the esophagus requiring removal and reintubation
Extubation	Accidental removal of the ETT requiring reintubation
Hypotension	Decrease in systolic blood pressure to <90 mmHg
Hypoxemia	A decrease in oxygen saturation below 90%
Laryngospasm	Adduction of vocal cords preventing passage of the ETT through the glottic inlet
Mainstem intubation	Radiographic identification of the tip of the ETT in a mainstem bronchus

*ETT*, endotracheal tube; *mmHg*, millimeters of mercury.

**Table 2:** First pass success by year over the 10-year period.

Academic Year	First Pass Success % (n)	95% CI
1 (2007–2008)	73.1% (250/342)	68.2% to 77.5%
2 (2008–2009)	73.9% (260/352)	69.0% to 78.2%
3 (2009–2010)	75.3% (220/292)	70.1% to 79.9%
4 (2010–2011)	79.8% (257/322)	75.1% to 83.9%
5 (2011–2012)	82.5% (320/388)	78.4% to 86.0%
6 (2012–2013)	85.2% (351/412)	81.4% to 88.3%
7 (2013–2014)	86.2% (367/426)	82.5% to 89.1%
8 (2014–2015)	89.1% (352/395)	85.6% to 91.8%
9 (2015–2016)	92.1% (348/378)	88.9% to 94.4%
10 (2016–2017)	92.4% (410/445)	89.5% to 94.5%

*CI,* confidence interval.

**Table 3:** Patients with adverse intubation events by year over the 10-year period.

Academic Year	Patients with Adverse Events % (n)	95% CI
1 (2007–2008)	22.5% (77/342)	18.4% to 27.2%
2 (2008–2009)	29.3% (103/352)	24.8% to 34.2%
3 (2009–2010)	25.0% (73/292)	20.4% to 30.3%
4 (2010–2011)	22.9% (74/322)	18.7% to 27.9%
5 (2011–2012)	23.5% (91/388)	19.5% to 27.9%
6 (2012–2013)	20.6% (85/412)	17.0% to 24.8%
7 (2013–2014)	22.8% (97/426)	19.0% to 27.0%
8 (2014–2015)	15.4% (61/395)	12.2% to 19.4%
9 (2015–2016)	15.3% (58/378)	12.0% to 19.3%
10 (2016–2017)	14.6% (65/445)	11.4% to 18.0%

*CI,* confidence interval.

**Table 4:** Adverse intubation events in Academic Year 1 versus Academic Year 10.

Specific Adverse Events	Academic Year 1 (2007–2008) %, (n = 342)	Academic Year 10 (2016–2017) %, (n = 445)	% Difference (95% CI)
Aspiration	1.8% (6)	0% (0)	−1.8% (−3.1% to −0.4%)
Cardiac arrest	0.6% (2)	0% (0)	−0.6% (−1.4% to 0.2%)
Cuff leak	0.6% (2)	0% (0)	−0.6% (−1.4% to 0.2%)
Dental injury	0.3% (0)	0% (0)	−0.3% (0.9% to 0.3%)
Dysrhythmia	0.3% (1)	0.2% (1)	−0.07% (−0.8% to 0.7%)
Esophageal intubation	4.4% (15)	0% (0)	−4.4% (6.6% to 2.2%)
Extubation	0.6% (2)	0.7% (3)	0.1% (−1.0% to 1.1%)
Hypoxemia	17.3% (59)	11.2% (50)	−6.0% (−11.0% to −1.1%)
Hypotension	0% (0)	2.9% (13)	2.9% (1.4% to 4.5%)
Laryngospasm	0% (0)	0.45% (2)	0.5% (−0.2% to 1.1%)
Mainstem intubation	2.6% (9)	0.9% (4)	−1.7% (−3.6% to 0.2%)
Total patients with adverse events	22.5% (77)	14.6% (65)	−7.9% (−13.4% to −2.4%)
Patients with 1 adverse event	17.5% (60)	12.8% (57)	−4.7% (−9.8% to 0.4%)
Patients with 2 adverse events	3.5% (12)	1.8% (8)	−1.7% (−4.0% to 0.6%)
Patients with ≥3 adverse events	1.5% (5)	0% (0)	−1.5% (−2.7% to 0.2%)

*CI,* confidence interval.

**Table 5:** Procedural characteristics in Academic Year 1 versus Academic Year 10.

Procedural characteristic	Academic Year 1 (2007–2008) %, (n = 342)	Academic Year 10 (2016–2017) %, (n = 445)	% Difference (95% CI)
Device used			
Direct laryngoscope	52.6% (180)	2.0% (9)	−50.6% (−56.1% to −45.2%)
Video laryngoscope	44.7% (153)	97.8% (435)	53.0% (47.6% to 58.5%)
Hyperangulated VL	100% (153/153)	7.4% (32/435)	−92.6% (−95.1% to −90.2%)
Standard Geometry VL	0% (0/153)	92.6% (403/435)	92.6% (90.2% to 95.1%)
GlideScope®	86.9% (133/153)	5.5% (24/435)	−81.4% (−87.2% to −75.7%)
C-MAC®	0% (0/153)	94.5% (411/435)	94.5% (92.3% to 96.6%)
Other VL	13.1% (20/153)	0% (0/435)	−13.1% (−18.4% to −7.7%)
Paralytic used			
Succinylcholine	40.3% (138)	54.0% (240)	13.6% (6.6% to 20.5%)
Rocuronium	59.1% (202)	45.8% (204)	−13.2% (−20.2% to −6.3%)
Sedative used			
Etomidate	90.4% (309)	81.6% (363)	−8.8% (−13.5% to −4.0%)
Ketamine	2.0% (7)	14.6% (65)	12.6% (9.0% to 16.2%)
Propofol	2.6% (9)	2.2% (10)	−0.4% (−2.6% to 1.8%)
Operator PGY			
PGY 1	17.3% (59)	13.7% (61)	−3.5% (−8.7% to 1.6%)
PGY 2	39.2% (134)	35.0% (156)	−4.1% (−10.9% to 2.7%)
PGY ≥3	43.6% (149)	51.2% (228)	7.7% (0.7% to 14.7%)
Rescue maneuvers			
Device switch	17.8% (61)	0.5% (2)	−17.4% (−21.5% to −13.3%)
Resident rescue	3.2% (11)	0.9% (4)	−2.3% (−4.4% to −0.3%)
Attending rescue	5.8% (20)	0.2% (1)	−5.6% (−8.1% to −3.1%)
SGD rescue	1.2% (4)	0% (0)	−1.2% (−2.3% to 0%)
CRIC rescue	0% (0)	0% (0)	0%

Other VL=McGrath®, Pentax Airway Scope®, Res-Q-Scope®.

*CI,* confidence interval; *VL,* video laryngoscope; *SGD*, supraglottic device; *CRIC,* cricothyrotomy; PGY, postgraduate year.
