# Supplementary material for: Improvement in the Safety of Rapid Sequence Intubation in the Emergency Department with the Use of an Airway Continuous Quality Improvement Program
Source: West J Emerg Med. 2019 Jun 3;20(4):610–8. doi: 10.5811/westjem.2019.4.42343 (PMC6625676; doi:10.5811/westjem.2019.4.42343)
Supplement: Supplementary file 1 [file wjem-20-610-s001.pdf]

Emergency Department Airway CQI Form

___/___/___
Date Form Filled Out

(Patient Sticker)
Name:
MRN:
Age:

Attending: ____________________
Resident: ____________________ (Specialty: _______)
PGY: I II III IV V Attending

○ Trauma → Mechanism_________________
Injuries____________________
○ Medical → Diagnosis__________________

Was there a FAILED intubation attempt *PREHOSPITAL*? ○ Yes ○ No
If **YES**, then: ○ NRB ○ BVM ○ LMA ○ Air-Q ○ King ○ Combitube ○ Cricothyrotomy ○ Esophageal Intubation

---

Did the patient **FAIL** BiPAP in the ED? ○ Yes ○ NA   If **YES**, for how long? ______ min

---

PREOX - AP OX

PREOX Device(s): ○ None

○ **NRB Face Mask:**	○ 5L/min	○ 10L/min	○ 15L/min	○ >15L/min	○ Flush 70	○ Other____
○ **Nasal Cannula:**	○ 5L/min	○ 10L/min	○ 15L/min	○ >15L/min		○ Other____
○ **BVM (spontaneous):**	○ 5L/min	○ 10L/min	○ 15L/min	○ >15L/min	○ Flush 70	○ Other____
○ **BVM (assisted):**	○ 5L/min	○ 10L/min	○ 15L/min	○ >15L/min	○ Flush 70	○ Other____
○ **Optiflow** *or* ○ **Vapotherm:**		○ 40L/min	○ 70 L/min	○ Other ______		
○ **BiPAP:**	IPAP_____cm / EPAP_____cm					

PREOX Position: ○ **Supine**
○ **Upright:** ⇨ ○ 30 degrees ○ 45 degrees ○ 60 degrees ○ 90 degrees

PREOX Duration: ○ 1 min ○ 2 min ○ 3 min ○ 4 min ○ 5 min ○ >5 min ⇨ ○ Estimated *or* ○ Timed

PREOX Efficacy: ***BEFORE*** PREOX: SpO_2 _______%
AFTER PREOX: SpO_2 _______%
○ ETO_2 Measured ETO_2 Achieved______%

AP OX Device: ○ None
○ **Nasal Cannula:** ○ 5L/min ○ 10L/min ○ 15L/min ○ >15L/min ○ Other____
○ **Optiflow** *or* ○ **Vapotherm:** ○ 40L/min ○ 70 L/min ○ Other______

Reason for Intubation (check **ONE**):
- ○ Airway Protection
- ○ Hypoxemic (Type I) Resp Failure
- ○ Hypercarbic (Type II) Resp Failure
- ○ Patient Control
- ○ Shock
- ○ Cardiac Arrest (○ Active CPR)

Intubation Technique
- ○ RSI   Cricoid Pressure: ○ Y ○ N
- ○ DSI
- ○ Sedation Only (○ light ○ deep)
- ○ Arrest/Near Arrest, NO MEDS
- ○ CRIC
- ○ Sed/No Meds ⇨ Switch to RSI

Intubation Drugs Used
- ○ Succinylcholine
- ○ Rocuronium
- ○ Atropine
- ○ Lidocaine
- ○ Other__________________
- ○ None
- ○ Etomidate
- ○ Ketamine
- ○ Propofol

Difficult Airway Factors

A. Difficult Situational Factors (check **ALL** that apply):
○ Patient Uncooperative ○ Right Equipment Not Available ○ Other_________ ○ None

---

B. Difficult Laryngoscopy/Intubation Factors (check **ALL** that apply):
○ Cervical Collar or Immobility ○ Small Mandible ○ Short Neck ○ Blood in Airway
○ Facial/Neck Trauma ○ Obesity ○ Restricted Mouth Opening ○ Vomit in Airway
○ Airway Edema ○ Large Tongue ○ Other _______________ ○ None

C. Difficult Physiologic Factors (check **ALL** that apply):
SEVERE: ○ Hypoxemia ○ Hemodynamic Instability ○ Metabolic Acidosis ○ RV Failure ○ Other __________ ○ None

---

D. Difficult Ventilation Factors (Face Mask & Supraglottic Device) (check **ALL** that apply):
○ Beard ○ Poor Mask Seal ○ Obesity ○ Age>55 ○ No Teeth
○ Stiff Lungs ○ Restricted Mouth Opening ○ Sleep Apnea ○ Disrupted Airway Anatomy ○ Other_________ ○ None

---

E. Difficult Surgical Airway Factors (check **ALL** that apply):
○ Neck Surgery/Mass/Radiation ○ Obesity ○ Limited Neck Access ○ Distorted Laryngeal Anatomy ○ Other_______ ○ None

Pre-Intubation Difficult Airway Assessment

**A. Situational Difficulty:**	○ Easy	○ Challenging	○ Difficult	**D. Ventilation Difficulty:**	○ Easy	○ Challenging	○ Difficult
**B. Intubation Difficulty:**	○ Easy	○ Challenging	○ Difficult	**E. Surgical Airway Difficulty:**	○ Easy	○ Challenging	○ Difficult
**C. Physiologic Difficulty:**	○ Easy	○ Challenging	○ Difficult	⇨ ***OVERALL DIFFICULTY:***	○ Easy	○ Challenging	○ Difficult

Over ⮌

Outcome

Lowest SpO_2 ***DURING*** Intubation:_______%

Attempt #1

Operator:______________

Device:_______________

Blade Type/Size:____________

Stylet: ○Standard ○GlideRite ○None

Bougie: ○Yes ○No (○Rescue/○Education)

Outcome (check **ONE**):

○**SUCCESS**

○**FAILED**

Reason for Failure:

○Esophageal Intubation

○Can't See Cords

○Can't Direct Tube

○Tube Wouldn't Pass

○Equipment Failure

Adverse Events:

○**NONE**

○Desaturation (<90%)

○Hypotension (<90mm)

○Aspiration

○Dysrhythmia

○Dental Injury

○Laryngospasm

○Cuff Leak

○Extubation

○Mainstem

○Pneumothorax

○Cardiac Arrest

○Other_________

Attempt #2

Operator:______________

Device:_______________

Blade Type/Size:____________

Stylet: ○Standard ○GlideRite ○None

Bougie: ○Yes ○No (○Rescue/○Education)

Outcome (check **ONE**):

○**SUCCESS**

○**FAILED**

Reason for Failure:

○Esophageal Intubation

○Can't See Cords

○Can't Direct Tube

○Tube Wouldn't Pass

○Equipment Failure

Adverse Events:

○**NONE**

○Desaturation (<90%)

○Hypotension (<90mm)

○Aspiration

○Dysrhythmia

○Dental Injury

○Laryngospasm

○Cuff Leak

○Extubation

○Mainstem

○Pneumothorax

○Cardiac Arrest

○Other_________

*If more than 2 attempts use an additional form(s)

Cormack-Lehane Grade of Laryngoscopic View

Grade 1 Grade 2 Grade 3 Grade 4

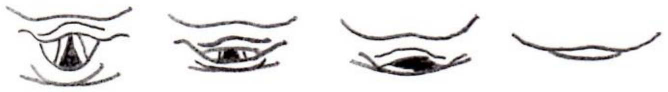

Full Cords Partial Cords Epiglottis Only Tongue Only

**DL View** (Attempt #):	_______	_______	_______	_______	ELM Used? ○Y ○N
**VL View** (Attempt #):	_______	_______	_______	_______	ELM Used? ○Y ○N

Tube Delivery Score
(*DIRECTING* the tube to the glottis)

○I Very Easy ○II Somewhat Difficult ○III Moderately Difficult ○IV Extremely Difficult

Tube Advancement Score
(*ADVANCING* the tube down the trachea)

○I Very Easy ○II Somewhat Difficult ○III Moderately Difficult ○IV Extremely Difficult

Stylet Withdrawal?
(to *FACILITATE* tube passage)

○**Yes** ○**No**

Video Laryngoscopy Section

If using Standard Geometry VL blade (C-MAC Mac/Miller, GlideScope-MAC or McGrath-MAC):

○ **Used as DL Only** Attempt#_____

○ **Used as VL Only** Attempt# _____

○ **DL to VL Switch** Attempt#_____

○ **VL to DL Switch** Attempt#_____

Lens Contamination (secretions, vomit, blood)

○ **None**

○ **Mild** (view minimally obscured)

○ **Moderate** (view partially obscured)

○ **Severe** (view completely obscured)

Lens Fogging (moisture)

○**None**

○**Mild**

○**Moderate**

○**Severe**

Please provide any important comments regarding the intubation:

____

____

BMI: _____

Weight: ____kg
